# Valorization of Eggshell Biowaste for Sustainable Environmental Remediation

**DOI:** 10.1038/s41598-020-59324-5

**Published:** 2020-02-12

**Authors:** Silvano Mignardi, Luana Archilletti, Laura Medeghini, Caterina De Vito

**Affiliations:** Department of Earth Sciences, Sapienza University of Rome, P.le A. Moro 5, I-00185 Rome, Italy

**Keywords:** Geochemistry, Pollution remediation

## Abstract

The management of large amounts of eggshell waste annually produced in the world is problematic as generally this material is only disposed at landfills with odor production and microbial growth. On the contrary, significant environmental and economic advantages could be obtained transforming this biowaste into new value-added products. Eggshell biowaste was the starting material for the synthesis of hydroxyapatite by a simple and sustainable procedure and applied for the removal of Co^2+^ from aqueous solutions. The effects of contact time and initial metal concentration were investigated in batch experiments. Eggshell-based hydroxyapatite (ESHAP) before and after Co^2+^ removal was characterized by X-ray diffraction and scanning electron microscopy. The process was rapid and reached equilibrium within 80 min. The removal efficiency was in the range 70–80% which is generally higher than other waste-derived adsorbents. Adsorption of Co^2+^ on the surface of ESHAP particles and ion exchange with Ca^2+^ resulting in the formation of a Co-phosphate are the main mechanisms of the metal removal. The conversion of eggshell waste to a low-cost adsorbent for the treatment of metal contaminated waters could contribute to a more sustainable and effective management of this biowaste.

## Introduction

The management of huge amounts of waste from the food processing industry is a challenging problem. The 3R principles (reduce, reuse and recycle) are the basis of the “Circular Economy”^1^. In this way, waste can be converted into valuable and useful resources improving both sustainable development and adequate waste management strategies^2,3^.

Eggshell is a typical example of product-specific waste in the food processing industry with utilizable parts still present in the waste^4^. Global egg production will increase to about 90 million tons by 2030^5–7^. As eggshells are considered useless, most of this waste is commonly disposed of in landfills without any transformation into useful materials^8,9^. However, the management of this waste requests adequate strategies to take into consideration increasing disposal costs, environmental concerns involving the risk of propagation of pathogens, unpleasant odor and availability of disposal sites^1,3,10^. Moreover, according to European Union regulations, eggshell is considered a hazardous waste^1,11^. Therefore, it is indispensable to find alternative ways to convert eggshell into valuable materials for further applications.

The shell weighs about 11% of the total mass of the egg and consists of calcium carbonate (94%), magnesium carbonate (1%), calcium phosphate (1%) and organic matter (4%)^12,13^. In this view, the reuse of eggshell waste in numerous applications would produce both environmental and economic advantages. The options for the valorization of this waste include the use of eggshell as a biological template for catalysis and antibacterial applications, the production of food additives, soil conditioners, the base material for cosmetics, pure calcium carbonate and biomaterials^1,7,12,14–16^. In particular, eggshell could be the starting material to prepare hydroxyapatite for the sustainable treatment of water polluted by toxic metals^17,18^. In addition, the conversion of eggshell waste into sorbent material for toxic metal remediation may reduce the mining impact on the natural reserves of phosphate rock that is included in the European Commission List of critical raw materials^19^.

In recent years many studies applied hydroxyapatite (HAP), Ca_10_(PO_4_)_6_(OH)_2_, for the remediation of toxic metal contaminated waters and soils^20–26^. In contrast to other toxic metals such as Cd, Pb, Cu, Zn, etc., the remediation of Co by HAP has been the topic of a limited number of studies^27,28^. Cobalt is largely present in the wastewaters from petrochemical, metallurgical and mining industries. Cobalt can cause neurotoxicological disorders, gastrointestinal symptoms and in chronic cases even cancer^28^. Moreover, ^60^Co is one of the most abundant radionuclides in nuclear wastewater^28,29^.

The main objective of this study is to evaluate the ability of eggshell-based HAP (ESHAP) in the removal of Co^2+^ ions from aqueous solutions. ESHAP sorption efficiency was studied by batch technique at different contact times and initial metal concentrations. The removal mechanisms were also investigated.

## Materials and Methods

### Preparation of ESHAP

All chemicals used were analytical reagent grade and purchased from Sigma Aldrich Chemical Company. The solutions were prepared with double distilled water. Waste eggshell were obtained from restaurants and bakeries in Rome, Italy. After washing of the eggshells several times with tap water, they were dried at 105 °C for 24 h in an oven. The eggshells were crushed in an agate mortar and then passed through a 100 µm sieve to obtain a homogeneous particle size. The prepared powder was stored in a desiccator until further experimentation. ESHAP was prepared as reported by Meski *et al*.^30^ adding the eggshell powder to a solution of HNO_3_ vigorously stirred (600–800 rpm) for 1 h at room temperature to obtain a solution of Ca(NO_3_)_2(aq)_ according to the reaction:

1 $${{\rm{CaCO}}}_{3}+2{{\rm{HNO}}}_{3}\to {\rm{Ca}}{({{\rm{NO}}}_{3})}_{2({\rm{aq}})}+{{\rm{CO}}}_{2}+{{\rm{H}}}_{2}{\rm{O}}$$

A solution of H_3_PO_4_ was slowly added dropwise to the solution of Ca(NO_3_)_2(aq)_, controlling the pH of the solution to 10 using a solution of NH_4_OH according to:

2 $$10{\rm{Ca}}{({{\rm{NO}}}_{3})}_{2}+6{{\rm{H}}}_{3}{{\rm{PO}}}_{4}+20{{\rm{NH}}}_{4}{\rm{OH}}\to {{\rm{Ca}}}_{10}{({{\rm{PO}}}_{4})}_{6}{({\rm{OH}})}_{2}\downarrow +20{{\rm{NH}}}_{4}{{\rm{NO}}}_{3}+18{{\rm{H}}}_{2}{\rm{O}}$$

After 1 h at ebullition temperature, the suspension was stored at room temperature for 24 h and then the precipitated ESHAP was collected by filtration.

### Adsorption experiments

The adsorption of Co^2+^ onto ESHAP was studied at 25 ± 2 °C in batch experiments. Synthetic Co^2+^ solutions were prepared by dissolving appropriate amounts of Co(NO_3_)_2_∙6H_2_O salt in double distilled water. The experiments were performed at various initial metal concentrations (50–500 mg L^−1^) in Nalgene beakers mixing 0.2 g of ESHAP with each Co^2+^ solution and stirring the suspensions and appropriate blank at 300 rpm. Co^2+^ solutions with a concentration of 100 mg L^−1^ were used to determine the kinetic constant. At predetermined time intervals in the range 0–180 min samples were drawn, filtered through 0.20 µm Nucleopore polycarbonate membrane filters and analyzed for Co^2+^ concentration. The optimized operating conditions were used in subsequent adsorption experiments. The adsorption mechanism was also investigated in detail by extending the contact time up to 6 weeks in some experiments. During the adsorption experiments, no pH control was imposed to simulate real conditions of industrial wastewaters treatment, where pH control is either not necessary or difficult to achieve. For the same reasons, a back-ground electrolyte was not used. All the experiments were conducted in duplicate and the mean values were used.

### Characterizations

The concentrations of Co^2+^ and Ca^2+^ in the filtrates from the adsorption experiments were analyzed by ICP-AES (Varian Vista RL CCD Simultaneous ICP-AES). Analytical detection limits for both Co^2+^ and Ca^2+^ were 0.03 mg L^−1^ and analytical errors were estimated in the order of 3%. The amount of Co^2+^ ions removed from the solutions and the percentage of metal adsorbed were calculated using the following equations, respectively:

3 $${{q}}_{{\rm{e}}}=({{C}}_{{\rm{i}}}-{{C}}_{{\rm{e}}})\times {V}/{M}$$

4 $${\rm{Percentage}}\,{\rm{of}}\,{\rm{metal}}\,{\rm{adsorbed}}\,( \% )=[({{C}}_{{\rm{i}}}-{{C}}_{{\rm{e}}})/{{C}}_{{\rm{i}}}]\times 100$$

were *q*_e_ (mg g^−1^) is the maximum amount of Co^2+^ adsorbed at equilibrium, the initial and final concentrations of Co^2+^ after equilibrium were shown as *C*_i_ and *C*_e_ (mg L^−1^), respectively, *M* is the mass of ESHAP (g) and *V* the total solution volume (L).

X-ray diffraction patterns of ESHAP before and after Co^2+^ adsorption were obtained in the range 5°–60° (2θ) with a step-size of 0.02° and counting time of 8 s using a Seifert MZIV automatic powder diffractometer with a Cu Kα radiation source, operating at 40 kV and 20 mA.

The morphological features of the samples were investigated by scanning electron microscopy (FEI-Quanta 400) with X-ray energy at 20 kV. Moreover, for each solid sample elemental analyses were carried out acquiring EDS spectra.

During the metal uptake, pH was measured by a pH 510 Eutec pH-meter.

### Adsorption kinetics

The Lagergren pseudo-first-order rate model^31^, the pseudo-second order model^32^ and the intraparticle diffusion model^33^ were used to analyze the adsorption kinetics of Co^2+^.

The linear form of pseudo-first-order equation is expressed as^29^:

5 $$\log (q{\rm{e}}-q{\rm{t}})=\,\log \,q{\rm{e}}-\frac{{k}_{1}}{2.303}t$$

where *q*_t_ (mg g^−1^) is the amount of Co^2+^ ions adsorbed at time *t* (min) and *k*_1_ (min^−1^) is the pseudo-first-order rate constant of adsorption^17^. The slope of the linear plot of log(*q*_e_ − *q*_t_) versus *t* provides the value of *k*_1_.

The pseudo-second-order model has the form^26^:

6 $$\frac{t}{q{\rm{t}}}=\frac{t}{q{\rm{e}}}+\frac{1}{{k}_{2}q{{\rm{e}}}^{2}\,}$$

where *k*_2_ (g mg^−1^ min^−1^) is the pseudo-second-order rate constant for adsorption. The value of *q*_e_ and *k*_2_ can be calculated from the plot of *t*/*q*_t_ versus *t*. Moreover, the initial adsorption rate *h* can be expressed as^26^:

7 $${h}={{k}}_{2}{{{q}}_{{\rm{e}}}}^{2}$$

Kinetics data were also analyzed using the intraparticle diffusion model. The involvement of intraparticle diffusion in the adsorption process is testify by a *q*_t_ versus the square root of time *t*^0.5^ linear plot^29^. The intraparticle diffusion could be the rate-limiting step of the process if the plot passes through the origin; otherwise some other mechanism may be also involved in the adsorption process^34,35^.

The intraparticle diffusion model is described as^34^:

8 $${{q}}_{t}={{k}}_{{\rm{i}}}{{t}}^{0.5}+{I}$$

where *k*_i_ is the intraparticle diffusion rate constant (mg g^−1^ min^−0.5^) and *I* (mg g^−1^) provides information about the thickness of the boundary layer.

### Adsorption isotherm

The adsorption models of Langmuir^36^, Freundlich^37^, Temkin^38^, and Dubinin–Radushkevich^39^ have been used to analyze the adsorption equilibrium data.

The Langmuir isotherm model assumes monolayer adsorption onto surface with a finite number of adsorption sites without interaction between the adsorbed molecules^35^. The model is expressed by the following equation^28^:

9 $$\frac{{C}_{{\rm{e}}}}{{q}_{{\rm{e}}}}=\frac{1}{{b}{{q}}_{{\rm{\max }}}}+\frac{{C}_{{\rm{e}}}}{{q}_{{\rm{\max }}}}$$

where *C*_e_ (mg L^−1^) is the concentration of Co^2+^ in solution at equilibrium, *q*_e_ (mg g^−1^) is the amount of metal adsorbed per unit mass of adsorbent, *b* (L mg^−1^) is the Langmuir constant related to energy of adsorption and *q*_max_ (mg g^−1^) is the maximum adsorption capacity^28^. The plot of *C*_e_/*q*_e_ versus *C*_e_ should be linear and provides the values of *q*_max_ and *b*. The dimensionless constant *R*_L_ provides information about the affinity between adsorbent and adsorbate and can be expressed as^40^:

10 $${R}_{{\rm{L}}}=\frac{1}{1+{b}{{C}}_{{\rm{i}}}}$$

where *b* is the Langmuir constant and *C*_i_ (mg L^−1^) is the initial concentration of Co^2+^ ions. The *R*_L_ values indicate the type of isotherm: irreversible (*R*_L_ = 0), favorable (0 < *R*_L_ < 1), linear (*R*_L_ = 1) or unfavorable (*R*_L_ > 1)^40^.

The Freundlich isotherm model is useful for describing multilayer adsorption in which the adsorbed molecules interact each other as it does not consider any saturation of the adsorbent by the adsorbate^40^. The linear form of the Freundlich equation is written as^40^:

11 $$\log \,{{q}}_{{\rm{e}}}=\,\log \,{{K}}_{{\rm{F}}}+\frac{1}{{n}}\,\log \,{{C}}_{{\rm{e}}}$$

where *K*_F_ (mg g^−1^) and *n* (g L^−1^) are the Freundlich constants referring to adsorption capacity and intensity, respectively. The Freundlich parameters can be calculated by plotting log*q*_e_ versus log*C*_e_. The value of the *n* constant is a measure of the linearity of adsorption: linear (*n* = 1); the adsorption is a chemical (*n* < 1) or physical (*n* > 1) process^41^.

The Temkin isotherm considers the effect of the heat of adsorption decreasing linearly due to coverage of the adsorbate and adsorbent interactions^42^. The linear form of the Temkin isotherm is^40^:

12 $${q}_{{\rm{e}}}=\frac{RT}{bT}ln{K}_{{\rm{T}}}+\frac{RT}{bT}ln{C}_{{\rm{e}}}$$

where *T* is the absolute temperature (K), *R* is the universal gas constant (8.314 J mol^−1^ K^−1^), *b*_T_ (J mol^−1^) is a constant related to the heat of adsorption and *K*_T_ (L g^−1^) is the equilibrium binding constant corresponding to the maximum binding energy^42^. A plot of *q*_e_ versus ln*C*_e_ enables the calculation of *b*_T_ and *K*_T_^40^.

The Dubinin-Radushkevich (D-R) model is generally applied to discriminate between physical and chemical adsorption^40^. The D-R model does not assume a homogeneous surface or constant adsorption potential^28^. This isotherm can be expressed in the linear form^41^:

13 $$\mathrm{ln}\,{{q}}_{{\rm{e}}}=\,\mathrm{ln}\,{{X}}_{{\rm{m}}}-{\beta }{{\varepsilon }}^{2}$$

where *X*_m_ (mg g^−1^) is the theoretical saturation adsorption capacity, *β* (mol^2^ J^−2^) is the activity coefficient related to mean adsorption energy and *ε* is the Polanyi potential, which is equal to^41^:

14 $${\varepsilon }={RT}\,\mathrm{ln}(1+1/{{C}}_{{\rm{e}}})$$

The *X*_m_ and *β* values can be obtained by a plot of ln*q*_e_ versus *ε*^2^. The constant *β* provides information about the mean adsorption energy, *E* (J mol^−1^), as follows^41^:

15 $${E}=\frac{1}{\sqrt{2{\beta }}}$$

The type of adsorption process is evaluated using the values of *E*: in the range between 8 and 16 kJ mol^−1^ the adsorption is due to ion-exchange, *E* < 8 kJ mol^−1^ the type of adsorption process is physical, *E* > 16 kJ mol^−1^ the adsorption occurs via chemisorptions^41^.

## Results and Discussion

### ESHAP characterization

**Figure 1:**
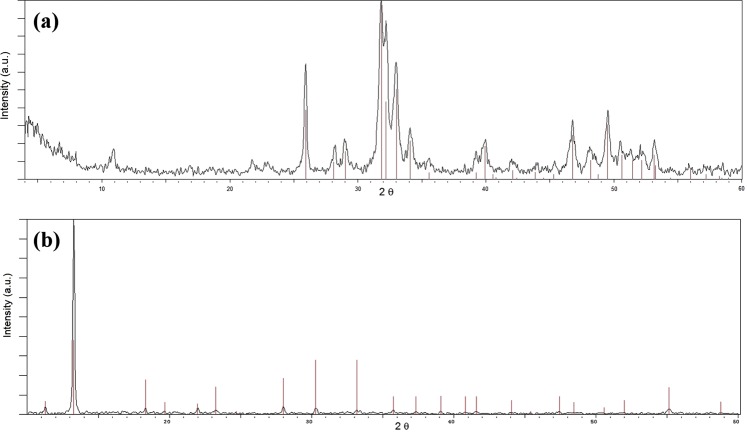
XRD patterns of ESHAP before (**a**) and after (**b**) the contact for 30 days with a solution containing 500 mg L^−1^ Co. For comparison purposes the corresponding crystallographic diffraction lines of standards are plotted.

XRD patterns of ESHAP are displayed in Fig. 1. As shown in Fig. 1a, the experimental data are in good agreement with HAP (JCPDS card no. 09-0432). After Co adsorption (Fig. 1b) ESHAP resulted completely transformed to a new mineral phase identified as pakhomovskyite [Co_3_(PO_4_)_2_∙8H_2_O] (JCPDS card no. 41-375), the Co-dominant analogue of vivianite^43^.

**Figure 2:**
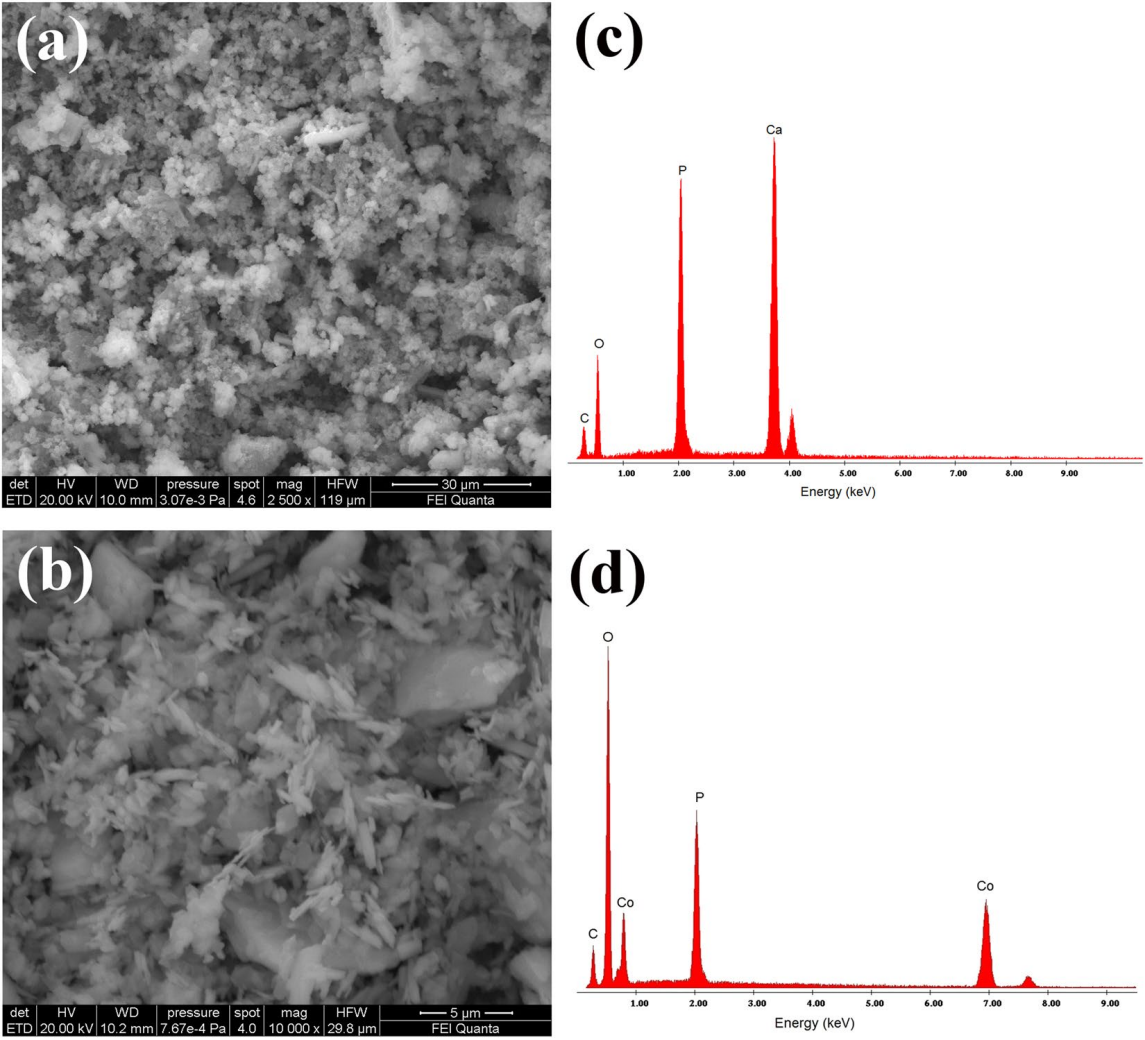
SEM micrographs of ESHAP before (**a**) and after (**b**) Co^2+^ removal and the corresponding EDS spectra (**c**,**d**).

**Figure 3:**
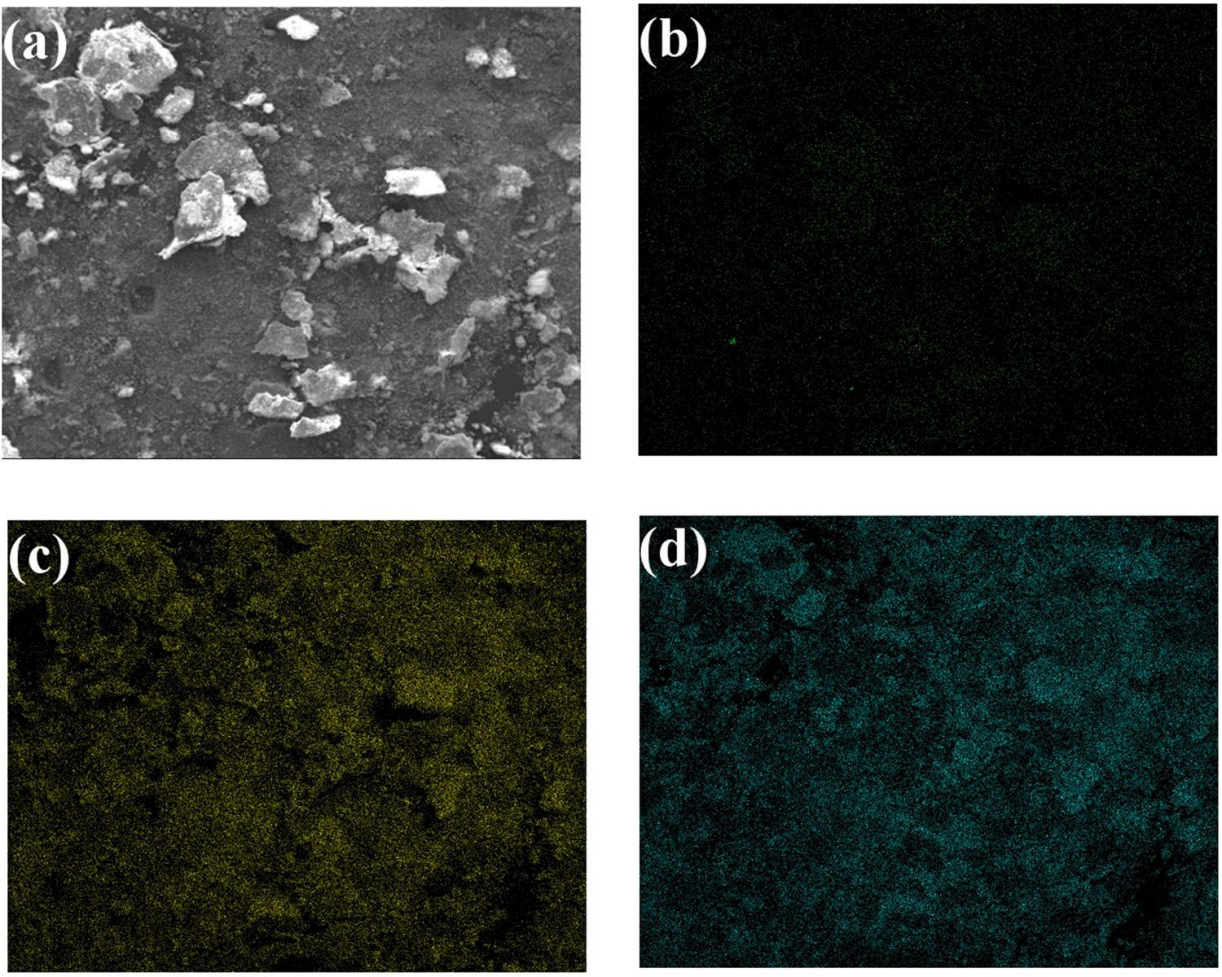
Imaging of Ca, P and Co in ESHAP after Co^2+^ removal; (**a**) BSE image; (**b**–**d**) imaging of Ca, P and Co.

The morphological features and external surface texture of ESHAP particles before and after Co^2+^ adsorption have been studied by SEM (Fig. 2). Before adsorption SEM analysis revealed a highly agglomerated ESHAP structure consisting of particles of sheet or flake-like structure with size in the range 10–20 µm (Fig. 2a). After the removal of Co^2+^ the morphology of the grains was significantly modified showing well-shaped tabular crystals, in agreement with the habit of natural pakhomovskyite, in the range 5–10 µm (Fig. 2b). This morphological change implies that during the removal of Co^2+^ from aqueous solution dissolution-precipitation phenomena occurred resulting in the crystallization of pakhomovskyite. Before Co^2+^ adsorption the components of ESHAP are Ca, P and O (Fig. 2c). The reaction with Co^2+^ caused the appearance of strong Co peaks in the spectra (Fig. 2d) and the almost complete disappearance of Ca peaks, clearly confirming the formation of pakhomovskyite in agreement with the XRD results. The distribution of the elements in ESHAP grains after Co^2+^ removal was obtained by EDS dot mapping (Fig. 3). The results showed that the entire surface of ESHAP grains is characterized by the presence of P. Co is uniformly distributed at high frequency, whereas Ca occurs only in a few areas with low frequency. In this view, the substitution of Ca by Co in the ESHAP grains is also suggested by the EDS dot mapping results.

### Effect of contact time

**Figure 4:**
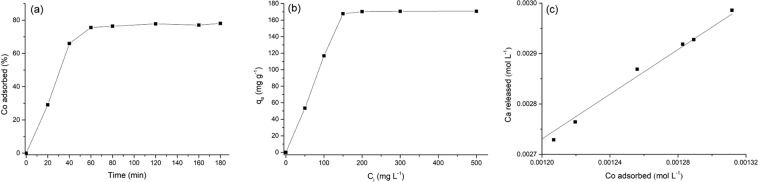
Effects of contact time (**a**) and initial metal concentration (**b**) on the removal of Co^2+^ by ESHAP; correlation between the amounts of Co^2+^ removed and the concentrations of Ca^2+^ released (**c**).

ESHAP removal efficiency shows a rapid increase up to about 75%, reaching the equilibrium in about 80 min with an adsorbed percentage of Co of 76.5% (Fig. 4a). The removal efficiency of Co^2+^ ions occurs in two steps: an initial fast stage in the first 60 min and a second slow stage until equilibrium. The initial fast Co^2+^ is attributed to the number and availability of vacant active sites on the surface of ESHAP grains which determined an increased concentration gradient between Co^2+^ in solution and metal ions on the adsorbent surface^44^. In the final stage (80–120 min) the observed less efficient removal of metal ions was due to the decrease of Co^2+^ concentration in the solution and the occupancy of active sites of ESHAP grains.

### Effect of initial Co^2+^ concentration

The Co^2+^ removal of ESHAP as a function of initial metal concentration is shown in Fig. 4b. The increase of the initial metal concentration leads to the increase of the amount of adsorbed Co^2+^ ions until the equilibrium was achieved. As a result of the occupy of all the active sites on ESHAP by Co^2+^ ions, then the adsorption of Co^2+^ seems to be independent of the initial metal concentration. Figure 4c shows the positive correlation existing between the amounts of Co^2+^ removed from the solution and the concentrations of Ca^2+^ released in the solution. However, the Co/Ca molar ratios were lower than 1 for all initial Co^2+^ concentrations. This result is consistent with a partial involvement of the ion exchange mechanism in the overall adsorption process accompanied by partial dissolution of ESHAP and consequent precipitation of Co-bearing phosphate on the surface of the grains as observed in previous studies^22^. A Co-containing phosphate is the result of the adsorption process.

### Kinetic study

**Figure 5:**
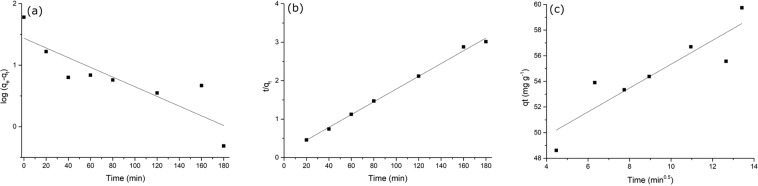
Pseudo-first-order kinetics (**a**), pseudo-second-order kinetics (**b**) and intraparticle diffusion plots (**c**) for adsorption of Co^2+^ onto ESHAP.

The adsorption process of Co^2+^ on ESHAP was deeply investigated considering the adsorption kinetics. In particular, pseudo-first-order, pseudo-second-order and intraparticle diffusion models were used. The relevant parameters are shown in Table 1, whereas Figs. 5a,b shows the linearized forms of the pseudo-first order model in Eq. (5) and the pseudo-second-order model in Eq. (6). The pseudo-second-order model well described the Co^2+^ adsorption onto ESHAP (*R*^2^ = 0.9964) (Table 1). In addition, the *q*_e,cal_ value agrees very well with the experimental *q*_e_ value. On the contrary, the value of *q*_e_ calculated using the pseudo-first-order model equation is only about 45% of the real value. Moreover, the correlation coefficient of the pseudo-first-order model (*R*^2^ = 0.7570) is worse than that of the pseudo-second-order model. Therefore, according to the assumption of the pseudo-second-order model, chemisorption involving valence forces by sharing or exchange of electrons between adsorbent and adsorbate^32^, could explain the overall adsorption process.

**Table 1:** Kinetics parameters of pseudo-first-order, pseudo-second-order and intraparticle diffusion models for Co^2+^ adsorption on ESHAP.

*q*_e,exp_ (mg g^−1^)	Pseudo-first-order model			Pseudo-second-order model				Intraparticle diffusion model		
	*q*_e,cal_ (mg g^−1^)	*k*_1_ (min^−1^)	*R*^2^	*q*_e,cal_ (mg g^−1^)	*k*_2_ (g mg^−1^ min^−1^)	*h*	*R*^2^	*k*_i_ (mg g^−1^ min^−1^)	*I*	*R*^2^
60.2	27.9	0.0182	0.757	60.3	2.30∙10^−3^	8.4	0.9964	0.9294	46.0443	0.8111

**Table 2:** Langmuir, Freundlich, Temkin and Dubinin-Radushkevich models parameters for the adsorption of Co^2+^ on ESHAP.

*q*_m,exp_ (mg g^−1^)	Langmuir model			Freundlich model			Temkin model			Dubinin-Radushkevich model			
	*q*_m,cal_ (mg g^−1^)	*b* (L mg^−1^)	*R*^2^	*K*_F_ (mg g^−1^)	n (g L^−1^)	*R*^2^	*b*_T_ (J mol^−1^)	*K*_T_ (L g^−1^)	*R*^2^	*X*_m,cal_ (mg g^−1^)	*b* (mol^2^ kJ^−2^)	*E* (kJ mol^−1^)	*R*^2^
424	457	0.0152	0.9894	18.6294	1.7388	0.9393	23.5466	1.8798	0.9923	279	4.0∙10^−5^	111.80	0.8467

**Figure 6:**
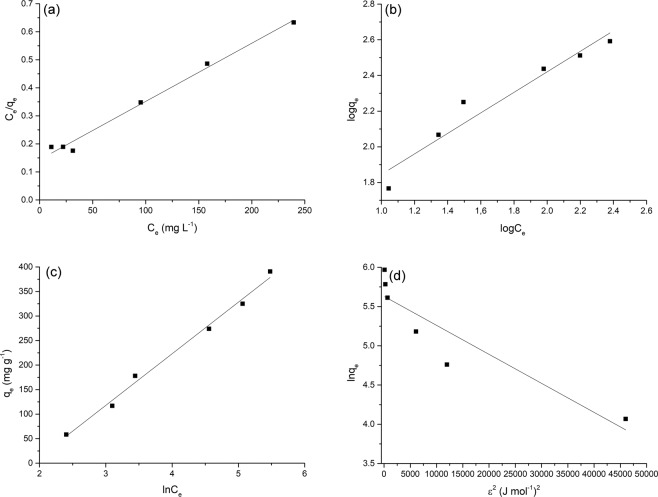
Isotherms for Co^2+^ adsorption onto ESHAP; (**a**) Langmuir, (**b**) Freundlich, (**c**) Temkin and (**d**) D-R models.

The intraparticle diffusion model was also applied to analyze the kinetic results. The plot of *q*_t_ versus *t*^0.5^ (Fig. 5c) is linear over the whole time range; however, the low value of the correlation coefficient (*R*^2^ = 0.8111) suggests that the intraparticle diffusion model only partly approximates the Co^2+^ adsorption process onto ESHAP. Moreover, the intraparticle diffusion cannot be considered the only rate-limiting step as the plot does not pass through the origin^34^. This result suggests that the internal diffusion was weak during the adsorption process; therefore, the Co^2+^ adsorption process is influenced by other mechanisms in addition to the intraparticle diffusion. In particular, intraparticle diffusion and surface adsorption could occur at the same time.

### Adsorption study

The experimental data fit better to the Langmuir and Temkin isotherm models (*R*^2^ = 0.9894 and 0.9923, respectively) than Freundlich (*R*^2^ = 0.9393) and D-R (*R*^2^ = 0.8467) models (Table 2, Fig. 6). The good regression coefficient of Temkin isotherm suggests the linear dependence of heat of adsorption at low or medium coverage. The applicability of the Langmuir model suggests the formation of an homogeneous and monolayer coverage of Co^2+^ ions on ESHAP grains.

Freundlich isotherm model adequately describes heterogeneous adsorption; anyway, the correlation coefficient was only partly satisfactory in the entire range of Co^2+^ concentrations. However, considering only the concentration range between 50 and 150 mg L^−1^, Freundlich model adequately describes the adsorption of Co^2+^ ions at low metal concentrations as proved by the linear plot log*q*_e_ versus log*C*_e_ and the excellent correlation coefficient (*R*^2^ = 0.9974).

Monolayer Co^2+^ adsorption on the surface of ESHAP grains is further indicated by the excellent agreement between the maximum adsorption capacity, *q*_m_, value of Langmuir model (457 mg g^−1^) and the experimental value (424 mg g^−1^)^45^. The different definition of the maximum adsorption capacity in the Langmuir and D-R models can explain the difference between the values also previously reported^46^. D–R model provides a value lower than that obtained from the Langmuir model as *X*_m_ represents the maximum adsorption capacity at the total specific micropore volume of the adsorbent^47^, whereas *q*_m_ at monolayer coverage.

The values of the separation factor *R*_L_ lying between 0.56 and 0.12 indicate favorable adsorption of Co^2+^ ions^41^. High affinity between ESHAP and Co^2+^ ions and favorable adsorption are indicated also by the values of the Freundlich constant *n* ranging between 1 and 10^41^.

The magnitude of the mean adsorption energy *E* (111.80 kJ mol^−1^), higher than 16 kJ mol^−1^, suggests that the adsorption occurs via chemisorptions^48^.

The Langmuir and Temkin models had a better fitting than Freundlich and D-R models indicating to the applicability of monolayer coverage of Co^2+^ ions on the ESHAP grains. In this view, the binding energy on the surface of grains is uniform and the metal ions do not interact or compete with each other.

Table 3 reports the comparison of the maximum adsorption capacity of ESHAP with that of other adsorbents for the removal of Co^2+^. Although the direct comparison of the removal capacity of different adsorbents is difficult due to different experimental conditions, the results of this study show that the removal capacity of ESHAP is higher than that of the adsorbents listed in Table 3.

**Table 3:** Comparison of Co^2+^ adsorption capacities for various adsorbents.

Adsorbents	*q*_m_ (mg g^−1^)	Reference
Almond green hull	45.5	^50^
Coir pith	12.82	^51^
Synthetic HAP	20.19	^52^
Hazelnut shell	13.88	^53^
Apricot stone	111.1	^54^
Lemon peel	22	^55^
Crab shell	20.47	^56^
Bentonite	7.3	^57^
ESHAP	457	This study

### Economic and environmental cost-benefit estimation

The concept of “circular economy” is based on recyclability, reusability and production of new materials from existing products. In this view, waste can be converted in value-added products enhancing both sustainable economic development and beneficial waste management. Indeed, the discharge of eggshell waste produced by an average egg processing plant in USA costs about US$ 100,000 per year^8^. On the contrary, the conversion of eggshell waste to ESHAP at industrial scale could produce an economical benefit at least 5 times higher than the cost of the conventional disposal methods^49^. At the same time, the reduction of the risk of spreading pathogens, reducing disposal costs and the production of a promising adsorbent for cost-effective wastewater remediation result in high environmental benefits strongly supporting the sustainability of the proposed method.

## Conclusions

The results of this study showed that eggshell biowaste is a suitable precursor material for the sustainable synthesis of adsorbent for the removal of Co^2+^ from wastewater. ESHAP removal capacity was evaluated at different contact time and initial Co^2+^ concentration. The adsorption process was well described by the pseudo-second-order kinetics model and only partly by the intraparticle diffusion model reaching equilibrium within 80 min. The Langmuir and Temkin models had a better fitting than Freundlich and D-R models with a maximum adsorption capacity of 457 mg g^−1^. The Co^2+^ removal mechanism involves surface adsorption on the ESHAP surface, ion exchange with Ca^2+^ and dissolution of the adsorbent followed by the precipitation of a new phosphate with formula Co_3_(PO_4_)_2_∙8H_2_O. The conversion of eggshell to a material for toxic metal remediation can contribute to the sustainable management of this biowaste.
